# Using Targeted Virotherapy to Treat a Resistant Ewing Sarcoma Model: From the Bedside to the Bench and Back

**DOI:** 10.1155/2014/171439

**Published:** 2014-01-12

**Authors:** Hesham Abdelbary, Christopher W. Brown, Joel Werier, John Bell

**Affiliations:** ^1^University of Ottawa, Division of Orthopaedic Surgery, Ottawa Hospital General Campus, 501 Smyth Road, Ottawa, ON, Canada K1H 8L6; ^2^Department of Orthopaedic Surgery, Queensway-Carleton Hospital, 3045 Baseline Road, Ottawa, ON, Canada K2H 8P4; ^3^University of Ottawa, Ottawa Regional Cancer Center Research Laboratories, 501 Smyth Road, Ottawa, ON, Canada K1H 8L6

## Abstract

Metastatic Ewing sarcoma (EWS) is often resistant to current multimodal chemotherapeutic regimens. Oncolytic virus therapy (OV) is a novel therapeutic platform whereby viruses can selectively infect as well as replicate in and kill tumor cells, while sparing normal tissues. The purpose of this study is to investigate the efficacy of the biotherapeutic oncolytic agent, vesicular stomatitis virus (VSVΔM51), to kill EWS cells that are resistant to conventional therapy. Our hypothesis is that systemic delivery of VSVΔM51 can demonstrate tumor-specific killing of resistant EWS cells, as well as a significant decrease of tumor burden in EWS bearing mice. *Methods*. A biopsy sample was obtained from a patient with metastatic EWS and was used to establish a novel EWS cell line. *In vitro* assays evaluated the oncolytic effect of vesicular stomatitis virus (VSVΔM51) on this cell line. EWS xenograft mice model bearing either lung or subcutaneous tumors was established to evaluate the antitumor specific oncolytic effect of VSVΔM51 after local and systemic delivery. *Results*. The established EWS cell line shared similar molecular and genetic traits to the patient's original tumor specimen. VSVΔM51 effectively infected and killed EWS cells *in vitro. In vivo,* VSVΔM51 selectively infected and killed EWS and led to significant delay in tumor growth. *Conclusion*. This study has been designed to implement a translational link between the bedside and the bench, where a specific challenging clinical scenario guided this basic science research. This research demonstrated that a sarcoma, which is resistant to current conventional standard therapies, is still susceptible to an alternative therapeutic platform, such as OV. Adding OV to the armamentarium of sarcoma treatment can enhance the future therapeutic approach towards these cancer patients.

## 1. Introduction

Sarcoma is a heterogeneous group of high-grade malignant tumors that originate from mesenchymal tissues. Over the past decade, medical therapies have reached a plateau with regard to changing the dismal coarse of this disease. Oncolytic virus therapy (OV) can offer a novel and a promising therapeutic platform for sarcoma patients. Phase I/II clinical trials of OV in carcinoma patients demonstrated remarkable safety profiles and notable clinical effects. No such trials have been attempted in sarcoma patients. In this paper, we demonstrate the ability of OV to target and kill resistant Ewing's sarcoma cells harvested from a patient who failed current conventional chemotherapy. This study provides preclinical evidence that OV can offer therapeutic benefit for patients with resistant sarcomas. Ultimately, similar studies need to be performed with different types of sarcomas in order to support the rational for initiating clinical trials of OV in sarcoma patients.

EWS is the second most common primary osseous malignancy in adolescence [1]. Over 85% of EWS cells possess an aberrant chromosomal translocation involving chromosomes 11 and 22, which produces the oncogenic chimeric fusion protein EWS/FLI1 [2].

EWS is a highly aggressive solid tumor that requires a multimodal therapeutic approach. The standard treatment regimen consists of multiagent chemotherapy for systemic control, in addition to local surgical resection and radiation. Although, over the past four decades, the 5-year overall survival of patients with nonmetastatic EWS has increased from 42% to 75% [1], this improvement has reached a plateau over the past decade. A more challenging clinical scenario is long-term control of advanced and metastatic disease. Up to 30% of EWS have macrometastasis at presentation and an overall 5-year survival of less than 20% [3]. In addition, current therapy-related morbidities, such as secondary malignancies and cardiac toxicity, contribute to over 20% of late mortalities in patients surviving for longer than 5 years after diagnosis [1]. Therefore, two important challenges currently facing EWS treatment are overcoming tumor resistance as well as enhancing tumor-targeted killing.

The root of tumor resistance lies in the complexity of cancer biology. It is the dynamic ability of the cancer cell to evolve and adapt to the host's anticancer environment that hinders the eradication of a cancer [4]. At the molecular level, these adaptive behaviors affect multiple pathways simultaneously such that cancer cells can inhibit programmed cell death, encourage autonomous cell growth, and promote the production of factors that enhance tumor vascularization and spread [5]. Cancer cells have also adopted mechanisms to downregulate inflammatory responses and evade adaptive immune surveillance directed against it [4]. It is this plasticity of the cancer that is thought to allow small subsets of cells to evade nontargeted therapy, resulting in the development of recurrent and refractory disease [6]. More robust tumor responses may result from therapies that disrupt or target more than one of the tumor's adaptive processes.

Oncolytic virotherapy (OV) is based on engineering viruses that selectively replicate in and kill cancer cells by exploiting their genetic abnormalities. Preclinical investigations have shown that interferon (IFN) is a key mediator of cellular antiviral defense mechanism and impairment of this pathway has been identified in 80% of tested human cancer cell lines [7]. OV are self-amplifying biotherapeutics that utilize a multimodal tumor-killing strategy either directly via lysis and induction of cytotoxicity, or passively by stimulating acute inflammatory reactions within the tumor bed causing thrombosis of its microvascular network and subsequently killing uninfected cancer cells within the tumor [8]. In addition, the stimulation of this intratumoral acute inflammation tends to break the tumor's immune tolerance, which subjects it to the host's adaptive immune response [5].

Over the past decade, promising preclinical research and human clinical trials using OV have focused on treating carcinomas [9]. There have only been a handful of studies testing the efficacy of OV in sarcoma models [10–12]. Morton et al. demonstrated the antitumor activity of Seneca Valley virus in neuroblastoma and rhabdomyosarcoma xenograft mice models [10]. Takakuwa et al. used Herpes Simplex virus (HSV) to treat intraperitoneal fibrosarcoma in immunocompetent mice [11]. HSV treatment resulted in cancer-free survival of 90% of the mice. Furthermore, attempts to reestablish fibrosarcoma by the injection of fresh tumor cells into previously cured mice were unsuccessful. These results provide a proof of principle that OV can induce an antisarcoma immune response in immune competent hosts, a result already described in models with carcinomas [5].

The purpose of this study is to investigate the efficacy of the biotherapeutic oncolytic agent, vesicular stomatitis virus (VSVΔM51), to kill EWS cells that are resistant to conventional therapy. Our hypothesis is that systemic delivery of VSVΔM51 can demonstrate tumor-specific killing of resistant EWS cells, as well as a significant decrease of tumor burden in EWS bearing mice.

## 2. Materials and Methods

### 2.1. Patient Recruitment

Approval for the study was obtained from both the Mount Sinai Hospital in Toronto and the Ottawa Hospital Research Ethics Board. Patients were recruited preoperatively by their respective surgeon and explained the nature of the study. Patients who agreed to participate signed an informed consent prior to their operation. Inclusion criteria consisted of patients undergoing surgery for the removal or biopsy of a confirmed or solid organ malignancy. The tumor may be primary or metastatic and the patient may have undergone previous adjuvant therapy or surgical resection. There are no incentives for participation by patient or treating physician. All demographic data obtained from patient and tissue samples remain confidential and labeled with a unique identifier to ensure patient privacy. The patient will have no contact with the research team unless they wish to speak to the principal or coinvestigators.

### 2.2. Virus

For production of virus used in animal experiments Vero cells were infected at an MOI of 0.01. Culture supernatants were collected 24 hours later and cleared by centrifugation and filtration through a 0.2 *μ* filter. Virus was pelleted and then banded on a continuous 5%–40% sucrose gradient made in PBS. Banded virus was extensively dialyzed against PBS, aliquoted, and stored at −80°C. Stocks were tittered on Vero cells. The virus used for infecting sarcoma tumor samples was the interferon inducing mutant strain of VSV, VSVΔM51, that has been further engineered to express the green fluorescent protein (GFP), or the red fluorescent protein (RFP) or the luciferase (Luc) reporter genes [13–15].

### 2.3. Specimen Processing

Samples of fresh sarcoma tumor are obtained from biopsies or resections. These samples are obtained from two tertiary sarcoma centers, Ottawa and Toronto, Canada. All samples are obtained and manipulated under sterile conditions and temporarily stored or transported at room temperature in Dulbecco's Modified Eagle's Medium (DMEM) containing 10% fetal bovine serum (FBS) (HyClone, Hudson, NH). Upon obtaining the tissue sample, the culture medium is refreshed and gentamicin is added to a final concentration of 0.01 mg/mL if there is concern of bacterial infection. The sample is then divided into separate 10 cm culture petri dishes. Samples were cut into 5 mm in size using standardized protocol [16]. A piece is left in the growth medium for cell culture at 37°C, another sample is frozen at −80°C for baseline control, and a third piece is incubated with VSVΔM51. Control samples consisted of normal adjacent tissue that is treated in parallel with the tumor sample.

Prior to incubating with VSVΔM51, the sample is washed in Phosphate Buffer Saline (PBS) and then covered in 500 *μ*L of DMEM containing 1 × 10^8^ particle forming units (PFU) of VSVΔM51 per mL. The virus is allowed to incubate for 45 minutes at 37°C and then 10 mL of DMEM with 10% FBS is added. Twelve hours after application of the virus the sarcoma cells are observed under fluorescent microscopy to confirm the presence of green fluorescent protein (GFP), thereby indicating successful transfection of the virus containing the gene for the GFP.

The portion of the biopsy sample that proliferated in culture underwent routine hematoxylin and eosin staining and was examined by a certified pathologist to confirm cellular atypia. Confirmed tumor cultures were then washed with PBS, and VSVΔM51 was diluted in PBS in multiplicity of infection (MOI) ratios of 1, 0.1 and 0.01, and was allowed to incubate at 37°C for 45 minutes prior to the addition of 10 mL of DMEM with 10% FBS. Samples were then observed under fluorescent microscopy at 12 h and 24 h after infection to confirm the presence of green or red fluorescent protein (GFP or RFP) or firefly luminescence from firefly luciferase using the IVIS, thereby indicating successful infection of the virus compared to controls. All strains used demonstrated same kinetics when tested with a single-step growth curves on Vero cells (data not shown) [14]. To confirm CD99 expression, cells were incubated with CD99 polyclonal antibody conjugated to fluorescein or corresponding isotype control and processed through a Fluorescent Activated Cell Sorter (FACS) (R&D Systems Catalog number FAB3968F, or isotype control Catalog number IC108F). Cell culture samples were also stained with a 1 : 1 volume of 0.8 mM trypan blue in PBS and observed within 30 minutes to indicate the incidence of cell death.

### 2.4. Animal Models

Female, 8- to 10-week-old CD1 nude were obtained from Charles River Laboratories (Wilmington, MA). All experiments were conducted with the approval of the University of Ottawa Animal Care and Veterinary Services. Intravenous (IV) administration of 100 *μ*L containing 4e8 PFU of virus was performed through the tail vein. Direct intratumoral injections of virus were performed using a 50 *μ*L volume, containing 4e8 PFU of virus, with a 27 gauge × 1/2′′ needle. Mice were monitored daily and euthanized at indicated time points or upon signs of morbidity by carbon dioxide narcosis. For the mouse tumor models, mice were either injected subcutaneously or intravenously with 1 e6 human Ewing sarcoma cells. Treatments began when subcutaneous tumors were visible and measure at least 13 mm^3^ or greater, whereas lung tumor bearing mice began treatments after 1 week of tumor implantation, a time determined from previous experiments. Tumor volumes in cubic millimeters were determined using digital calipers and the formulae (lager measurement/2) × (smaller measurement) [2]. End-points that dictated the sacrifice of mice included tumor volume >1800 mm^3^, skin ulcerations, weight loss <4.0 g, and hind limb paralysis. Three animals were used and each animal had bilateral tumors and the entire experiment was repeated.

### 2.5. *In Vivo* IVIS Imaging

Mice were injected with 200 *μ*L (10 mg/mL in PBS) of d-luciferin intraperitoneally (10 mg/mL in PBS) (Molecular Imaging Products Company, Ann Arbor, MI) for Firefly luciferase imaging. Mice were anesthetized under 3% isofluorane (Baxter Corp., Deerfield, IL) and imaged with the *in vivo* imaging system 200 Series Imaging System (Xenogen Corporation, Hopkinton, MA). Data acquisition and analysis was performed using Living Image v2.5 software. For each experiment, where appropriate, images were captured under identical exposure, aperture and pixel binning settings, and bioluminescence is plotted on identical color scales.

### 2.6. Immunohistochemistry

Tissues were harvested as described, placed in OCT mounting media (Tissue-Tek, Sakura Finetek, Torrance, CA, USA) and sectioned in 4 *μ*m sections with a microtome cryostat (Microm HM500 OM Cryostat). Sectioned tissues were fixed in 4% paraformaldehyde for 20 minutes and used for hematoxylin and eosin (H&E) staining or immunochemistry (IHC). IHC was performed using reagents from a Vecastain ABC kit for rabbit primary antibodies (Vector Labs, Burlingame, CA), according to instructions provided. Primary antibodies used were polyclonal rabbit antibodies against VSVΔM51 (gift of Earl Brown). Briefly, endogenous peroxidase activity was blocked by incubating with 3% H_2_O_2_ followed by blocking of nonspecific epitopes with 1.5% normal goat serum, then by blocking with avidin and biotin. PBS washes were performed between all blocking and incubating steps. Sections were incubated with anti-VSVΔM51 antibody (1 : 5000, 30 minutes) followed by anti-rabbit biotinylated secondary antibody. The avidin:biotinylated enzyme complex was added and the antigen was localized by incubation with 3,3-diaminobenzidine. Sections were counterstained with hematoxylin. To assess apoptosis sectioned tissues were processed as previously described with Anti-Active Caspase 3 (1 : 2000, 60 minutes). Slides were scanned on a Nikon Coolscan.

### 2.7. Analysis of Tumor Perfusion

Mice were injected intravenously with 100 *μ*L of a 50% solution of 100 nm diameter orange fluorescent microspheres (Molecular Probes, Burlington, Canada). Five minutes later, animals were sacrificed and the tumors harvested, covered in OCT media, and then immediately snap frozen on dry ice and then placed at −80°C until ready for sectioning as previously described. Tumor perfusion was analyzed by visualizing fluorescent microspheres in the vasculature of 10 *μ*m unfixed frozen sections using a ScanArray Express microarray scanner with a standard Cy3 laser (Packard Bioscience).

### 2.8. Statistical Methods

Data are represented as means ± standard error. *P* values for tumor volumes in animal studies were determined using nonpaired student's *t*-test. Virus quantifications from plaque assays were log transformed before statistical analysis and plotting.

## 3. Results

### 3.1. Case Report

A twenty-five-year-old male presented with a 6-month history of right shoulder pain. Initial plain films demonstrated a pathological fracture of the proximal humerus. Systemic staging confirmed metastatic disease of the lung, spine, sacrum, and femurs. A biopsy sample from the proximal humerus lesion was obtained. The histology and IHC analysis confirmed the diagnosis of EWS. The patient subsequently received neoadjuvant treatment consisting of four monthly rounds of vincristine, adrinomycin and cyclophosphimide that were well tolerated, along with external beam radiation to right proximal humerus and sacrum. Follow-up CT chest during the course of treatment showed mixed interval response. Four months after diagnosis, the patient presented with neck pain. Magnetic resonance imaging (MRI) showed a mass compressing the spinal cord at the level of C2. Subsequently, he underwent an urgent cervical spine laminectomy, decompression, and fusion. The histology and IHC analysis performed on the c-spine lesion confirmed EWS. Six months after diagnosis, CT chest detected enlarging pulmonary masses and bilateral chest tubes were placed to treat the pulmonary edema. Despite conventional treatment, patient succumbed to the disease 6 months after diagnosis.

### 3.2. Primary EWS Tissue Specimen Is Susceptible to VSVΔM51 Infection

**Figure 1:**
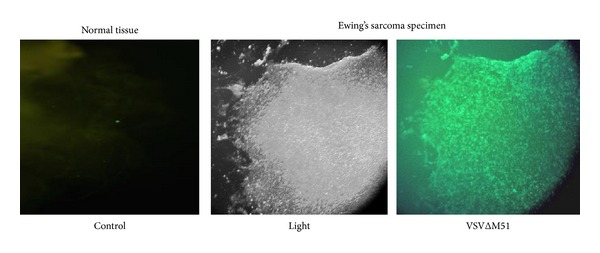
VSVΔM51 infects primary human Ewing sarcoma specimen obtained from the proximal humerus lesion. The tumor specimen was inoculated with 1 × 10^6^ pfu/mL of VSVΔM51-GFP. GFP expression was monitored using a fluorescent microscope 24 hrs after inoculation and the green fluorescence represents infected cells. Normal adjacent muscle control tissue is resistant to infection.

**Figure 2:**
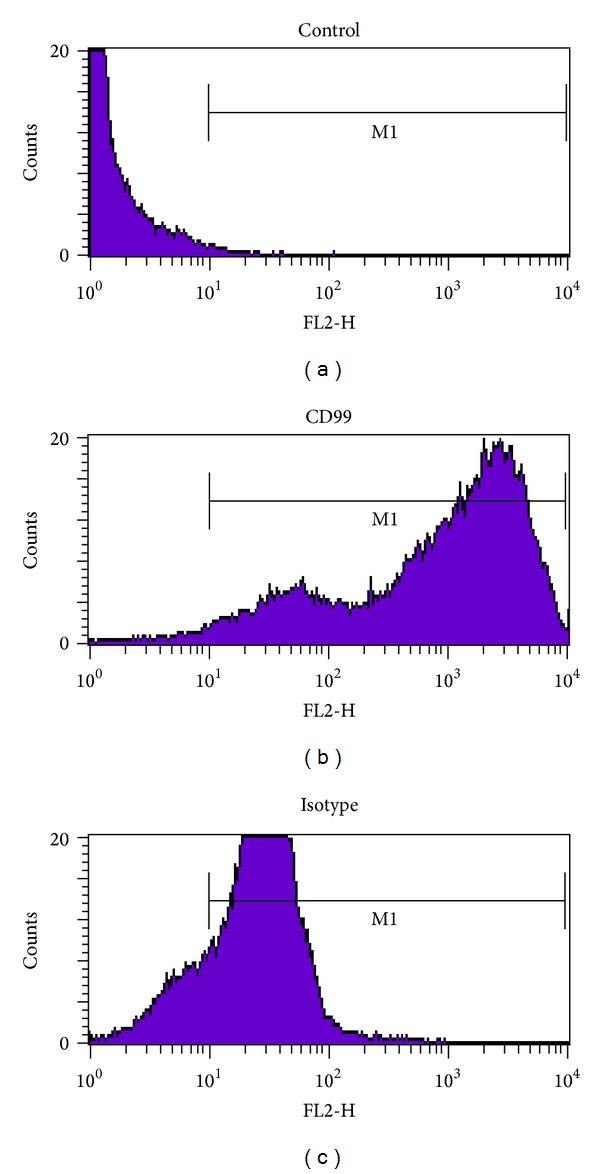
Cell line derived from the EWS biopsy is CD99 positive. After several passages, the established cell line was incubated with CD99 polyclonal antibody conjugated to fluorescein or corresponding isotype control and processed through a Fluorescent Activated Cell Sorter (FACS) as per manufacturers recommendations (R&D Systems Catalog number FAB3968F, or isotype control Catalog number IC108F).

To determine the susceptibility of EWS cells to VSVΔM51 infection, a representative tissue sample obtained from the patient's proximal humerus lesion was incubated with VSVΔM51 encoding the green fluorescent protein (GFP) gene. After 24 h, abundant GFP expression throughout the whole specimen was detected by fluorescent microscopy, which correlates with robust viral infection and replication whereas normal control tissue only has background fluorescence (Figure 1). To establish a permanent EWS cell line from the obtained biopsy specimen, primary cells were grown off the tumor specimen in tissue culture and were subjected to continuous weekly passaging. Successful passages promoted and maintained the growth of tumor cells. Flow cytometry was then used to identify the percentage of these passaged cells that were positive for CD 99, a surface marker commonly expressed by EWS cells (Figure 2). Cytogenetic testing was also performed on these isolated tumor cells. This data was published in our previous work, Maire et al. 2008, which confirmed that the immortalized EWS cell line expressed a unique t(19,22) translocation identical to that of both the humerus biopsy preadjuvant therapy and to a cervical lesion that was post adjuvant therapy [17].

### 3.3. VSVΔM51 Infects and Kills EWS Cells Grown from the Established Cell Line

**Figure 3:**
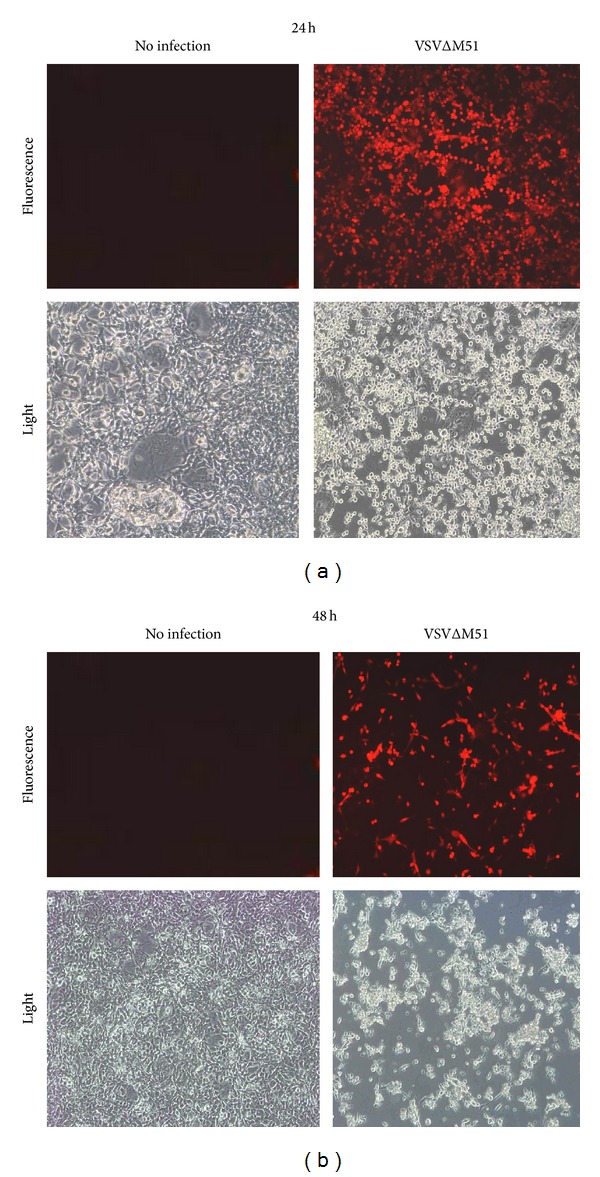
VSVΔM51 infects and kills EWS cells established from the patient's tumor specimen. EWS cells were inoculated with VSVΔM51-RFP at MOI 0.01. Viral replication was assessed at 24 h and 48 h by fluorescent microscopy for RFP expression. Light microscopy was used to assess cell death after infection. Robust infection was noted at 24 h and massive cell death was confirmed qualitatively by characteristic cytopathic rounding and detachment of cells off the culture dish at 48 h.

**Figure 4:**
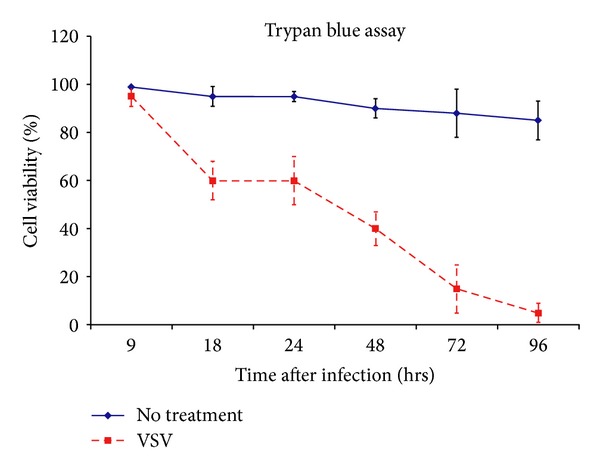
EWS cell death is assessed quantitatively by trypan blue assay. EWS cells were inoculated with VSVΔM51 at MOI 0.1. Viability of tumor cells at 96 h after infection is less than 10%.

To verify if the established EWS cell line was susceptible to VSVΔM51 infection, a confluent monolayer of these cells was inoculated with virus at a low multiplicity of infection (MOI) of 0.01. VSVΔM51 encoding red fluorescent protein, RFP, was used to monitor its replication. After 24 h, fluorescent microscopy showed RFP expression across the whole cellular monolayer. At 48 h, light microscopy confirmed significant cell death caused by VSVΔM51 infection. The extent of cytotoxicity also correlated with the decreased intensity in RFP expression at 48 h (Figure 3). Using trypan blue, a quantitative assay was used to measure the amount of tumor cell death caused by VSVΔM51 at various time points. Approximately 50% of tumor cellular death occurred 48 h after infection and less than 10% of tumor cells were viable 96 h after infection (Figure 4), highlighting the virulence of VSVΔM51. Additional FACS analysis of VSVΔM51 infected EWS tumor cell samples incubated with 5 *μ*L of 50 *μ*g/mL of Propidium Iodine (PI), a fluorescent stain that penetrates the cell membrane indicating cell death, demonstrated similar tumor cell death results (data not shown).

### 3.4. *In Vivo*, VSVΔM51 Strictly Infects the Tumor after Systemic Administration

**Figure 5:**
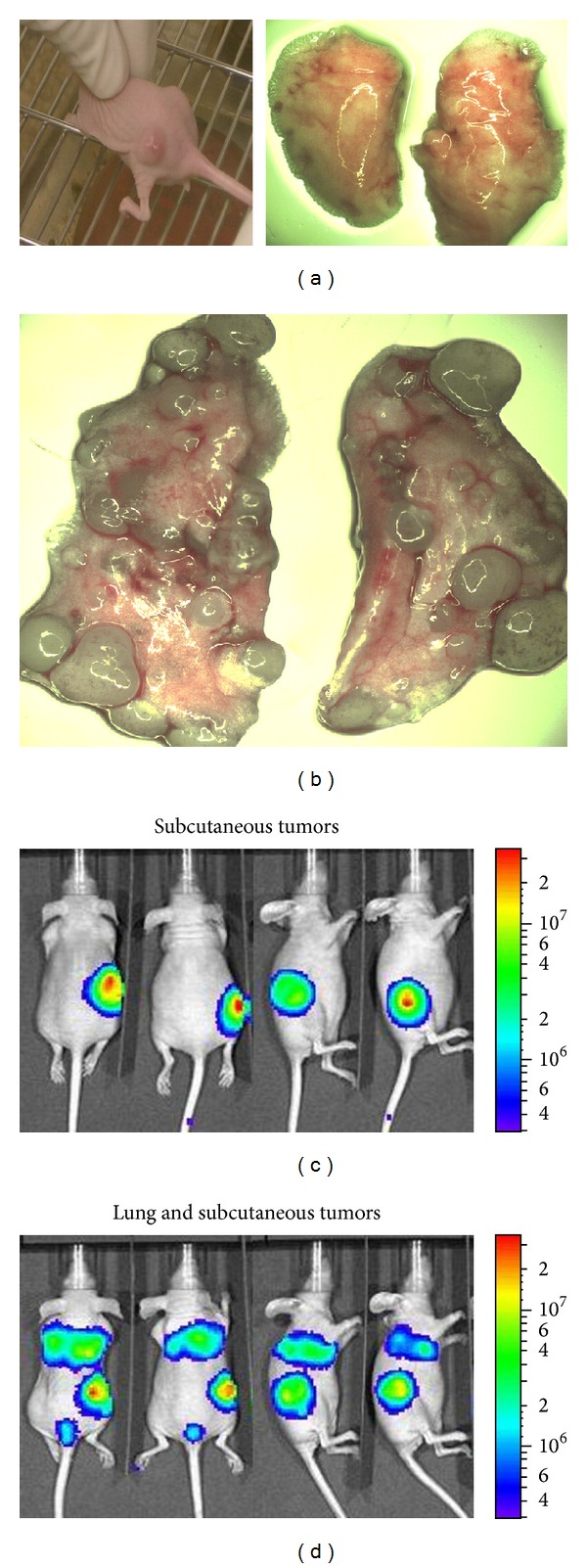
VSVΔM51 shows tumor-specific replication after its systemic delivery in nude mice harboring subcutaneous and lung tumors. In one group (a), a solid tumor mass developed in the hind-foot region a week after the subcutaneous inoculation of EWS cells. No tumor nodules were noted on gross inspection of the lungs (a). The second cohort of mice (d) was inoculated with EWS both subcutaneously and intravenously. Numerous tumor nodules were noted bilaterally on gross inspection of the lungs (b). After tumor growth in both groups, VSVΔM51-Luc was administered intravenously. At 72 h after treatment, *In vivo* Imaging System (IVIS) was used to assess viral replication in live animals. The luciferase signal is noted only at the tumor site in both groups ((c)-(d)).

Two xenograft EWS mouse models were established to demonstrate that VSVΔM51 selectively replicates in tumors and not in adjacent normal tissues or organs. In the first model, solid tumors developed in the hind limb after subcutaneous injection of EWS cells (Figure 5(a)). The second model harbored subcutaneous tumors as well as lung tumor nodules after subcutaneous and intravenous injection of EWS cells, respectively (Figure 5(b)).

**Figure 6:**
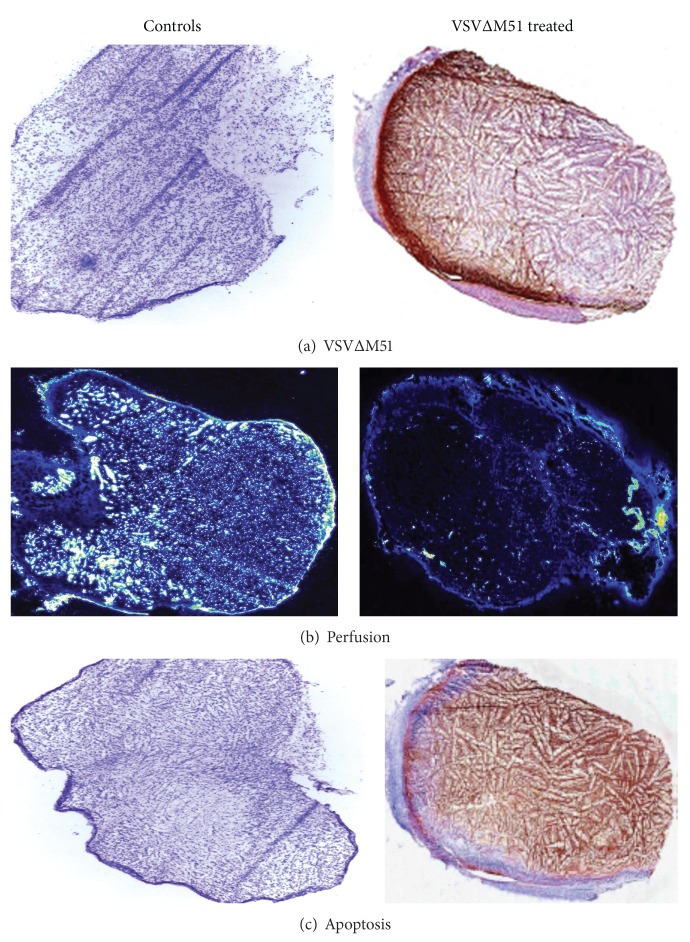
VSVΔM51 treatment leads to apoptosis of tumor cells and profound loss of tumor vasculature. At day 5 after treatment, subcutaneous tumors were harvested. IHC performed on tumor-frozen sections shows robust VSVΔM51 spread (a) within the tumor mass with corresponding tumor apoptosis (c). Microperfusion studies also indicated significant microvascular compromise postviral treatment (b). Normal controls are unaffected.

Each model then received a single intravenous injection of VSVΔM51 that was engineered to express the firefly luciferase protein. This protein allowed for *in vivo* monitoring of viral replication. At 72 h post-VSVΔM51 injection, the luciferase signal was only detected at the subcutaneous tumors in mice injected with the EWS subcutaneously as well as at the lung fields in the model that received EWS cells intravenously (Figures 5(c)-5(d)). Immunohistochemical staining of frozen tumor sections confirms the abundant presence of VSVΔM51 antigen after viral treatment but not in the adjacent normal tissue (Figure 6(a)). These results provide a proof of principle that VSVΔM51 is able to selectively infect and replicate at the tumor site despite its systemic administration.

### 3.5. VSVΔM51 Initiates Several Oncolytic Strategies *In Vivo* in EWS

**Figure 7:**
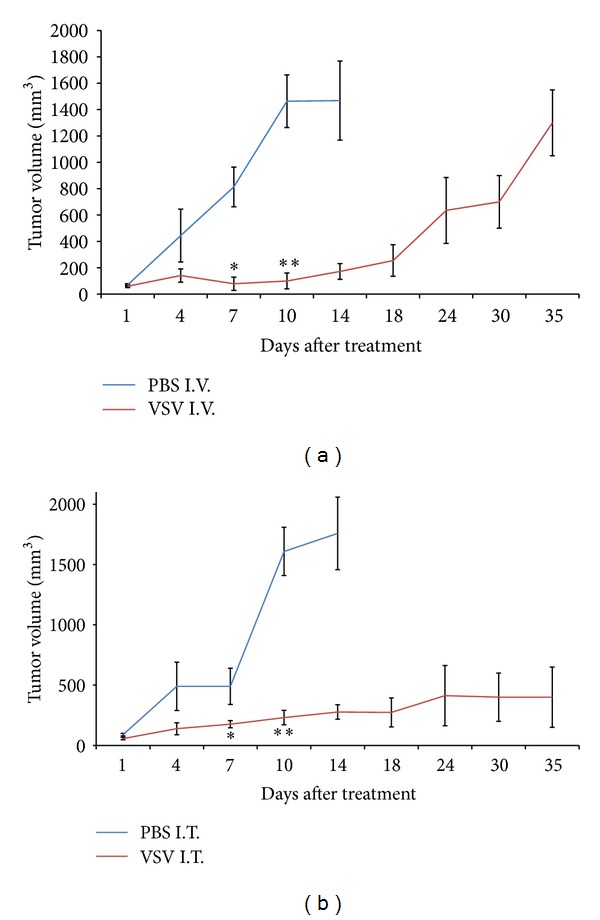
VSVΔM51 treatment significantly reduces tumor growth after local and systemic administration. (a)-(b) A single VSVΔM51 injection was administered either intravenously (I.V.) (a) or intratumoral (I.T.) (b). Tumor size was monitored over a period of time. Mice were sacrificed based on a preset tumor volume decided upon with animal care. Error bars represent standard error of the mean *P < 0.05, **P < 0.005 (Student's *t*-test). *N* = 4 mice per group. Note VSV in legend represents VSVΔM51.

Microperfusion studies performed on subcutaneous tumors harvested from VSVΔM51 treated mice also indicated profound loss of blood flow to the tumor (Figure 6(b)). This phenomenon has been well described by Breitbach et al. in carcinoma models [8]. VSVΔM51 treatment also led to tumor cell death that was confirmed by the abundant presence of the apoptotic marker active caspase 3 (Figure 6(c)). Also, a significant delay in tumor growth (*P* < 0.005) was subsequently observed after either the intratumoral or intravenous routes of VSVΔM51 administration (Figure 7). Intratumoral treatment resulted in a more pronounced and sustained suppression of tumor growth compared to intravenous treatments.

### 3.6. VSVΔM51 Infects Multiple Types of *Ex-Vivo* Human Sarcomas

**Figure 8:**
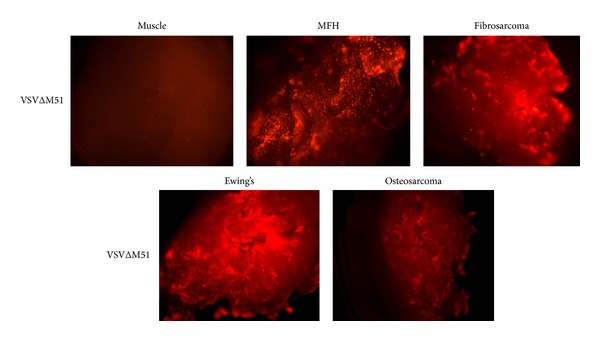
VSVΔM51 infects multiple types of *ex-vivo* human sarcomas. Approximately 50% of the *ex-vivo* specimens tested are susceptible to VSVΔM51 infection.

To determine if other types of sarcomas were susceptible to VSVΔM51 infection, various high-grade human sarcoma biopsy samples were tested. VSVΔM51 infection was confirmed qualitatively by the presence of fluorescent protein expression (Figure 8). Over 50% of the total number of tested tumor specimens were susceptible to VSVΔM51 infection (Table 1). However, certain sarcoma subtypes such as liposarcoma and fibrosarcoma showed less than 50% susceptibility. These poor results might relate to the nature of the matrix surrounding these tumor cells, which can act as a physical barrier to viral spread.

## 4. Discussion

Long-term control of advanced and metastatic sarcoma remains difficult despite advancements in anticancer therapies. One of the limitations of current conventional treatment regimens is the utilization of a targeted approach to kill tumor cells. Preclinical research has demonstrated the ability of oncolytic viruses to harness multiple modes of action to kill tumors. Improved understanding of tumor and virus biology and advancements in genetic engineering is allowing the use of diverse virus species as tumor-selective killing agents. Most preclinical studies have focused on carcinoma models. Some of these studies have demonstrated VSVΔM51 to be a potent oncolytic viral agent, killing malignant carcinoma cells while leaving normal tissues unharmed [7]. Our research is the first to highlight the antitumor effect of VSVΔM51 against a resistant EWS model.

In this research, our objective was to test the tumor-specific killing ability of VSVΔM51 against EWS model that was resistant to conventional therapies. This sarcoma model was developed by directly translating a therapeutic challenge from the clinical setting to the laboratory. A novel EWS cell line was established from a primary tumor specimen of a patient who failed conventional therapy. We hypothesized that VSVΔM51 can demonstrate tumor-specific killing of resistant EWS after systemic delivery, as well as a significant decrease of tumor burden in EWS bearing mice.

The resistance of our novel cell line could be that it is not an EWS-FLI1 fusion type, as the most common subtype, type 1, being associated with a favorable prognosis Ewing sarcomas with EWS-FLI1 and EWS-ERG gene fusions has similar clinical phenotypes supporting the notion that our cell line is perhaps representative of the less favorable subtypes, and perhaps the clinical result of our study could translate to other the more aggressive resistant types of EWS [17–19]. *In vitro* analysis proved the ability of VSVΔM51 to infect Ewing cells and to spread effectively across the cellular monolayer leading to 50% cell death in 18 h. Since VSVΔM51 replication is extremely sensitive to cellular innate production of IFN, we suspect that our model of EWS cells possessed a defective IFN response mechanism as this abnormality in cellular innate immunity has been described in other tumor cell types and has been proposed as a strategy used by tumor cells to facilitate their escape from immune surveillance [13].

Our research also presents strong evidence for tumor-specific replication of VSVΔM51 after systemic delivery. *In vivo* imaging confirmed that VSVΔM51 replicated specifically at the tumor site whether located subcutaneously or in the lung parenchyma. This naturally inherent tumor targeting ability of VSVΔM51 makes it a favorable oncolytic viral agent. IHC analysis has shown that in addition to inducing tumor cell death directly by cytolysis, oncolysis was also mediated by triggering apoptosis. This apoptosis is activated by the release of cytokines from infected and dying tumor cells, as well as from hypoxic cells resulting from damaged tumor microvasculature postviral treatment [4]. VSVΔM51 infection indirectly influences the tumor by attracting neutrophils and other inflammatory cells to the tumor mass [20]. An acute inflammatory reaction ensues, activating the coagulation cascade within the tumor microvasculature that results in ischemic central necrosis of the tumor. Breitbach et al. have demonstrated that neutrophil depletion or anticoagulants tend to reduce the OV tumor killing in a number of *in vivo* carcinoma models [8]. This multimodal attack against the tumor provides OV with an advantage over current conventional anticancer therapies. Indeed, in our *in vivo* model, VSVΔM51 caused significant delay in tumor growth prolonging survival. However, a weakness in our study is the inability to demonstrate a cure. Previous research has demonstrated the importance of the adaptive immune response in determining cures with OV. T-lymphocytes in particular play a central role in the development of the oncolytic virus/VSVΔM51 mediated adaptive antitumor immunity [21, 22]. The lack of cures in our animal model is likely as a result of using an immune compromised mouse model in order to support the growth of human EWS cells.

There are over twenty viral species that have been tested for their ability to act as oncolytic agents. Each of these OV's holds a vast array of genetic information and utilizes different strategies to infect and kill cells. A thorough survey for more potent tumor-selective viruses may identify novel mechanisms of viral mediated tumor killing, thus leading to better cancer killers. An effective screening tool that can be utilized to identify the most appropriate oncolytic viral agent against a specific sarcoma type is to test the susceptibly of primary fresh *ex-vivo* tissue specimen to viral infection. This method can serve as a quick and efficient screen to tailor the appropriate OV for a specific tumor type. Our pilot screen using this technique showed over 50% susceptibility to VSVΔM51 infection.

Currently, basic science and clinical research are experiencing a renaissance in cancer virotherapy. Replication competent, oncolytic virus based biotherapeutics have been shown to be safe platforms for the targeted treatment of cancer. The expanding understanding of virus and tumor biology coupled with advancements in recombinant genetic technology has provided a new tool for the treatment of cancer. Unfortunately, the therapeutic potential of oncolytic viruses against sarcoma is greatly under researched. Future preclinical sarcoma research should invest more efforts to explore the therapeutic potential of OV, in order to facilitate its translation to the sarcoma clinic.

## Figures and Tables

**Table 1:** VSVΔM51 infects multiple types of *ex vivo* human sarcomas. Approximately 50% of the *ex vivo* specimens tested are susceptible to VSVΔM51 infection.

Sarcoma type	No. of biopsy samples	Susceptible to VSVΔM51	%
Malignant fibrohistiocytoma	17	11	65
Osteosarcoma	14	9	64
Liposarcoma	13	4	31
Chondrosarcoma	5	4	80
Fibrosarcoma	4	1	25
Synovial sarcoma	3	2	67
Ewing's sarcoma	3	2	67
Dermatofibrosarcoma protuberans	1	1	100
Neuroblastoma	1	0	0
Hemangiopericytoma	1	1	100
Peripheral nerve sheath tumor	1	1	100
Myxoma	2	0	0
Leiomyosarcoma	2	1	50
Chordoma	1	1	100

Total	68	38	56
